# Enhanced YOLOv8 for Robust Pig Detection and Counting in Complex Agricultural Environments

**DOI:** 10.3390/ani15142149

**Published:** 2025-07-21

**Authors:** Jian Li, Wenkai Ma, Yanan Wei, Tan Wang

**Affiliations:** 1 School of Biology and Food Engineering, Fuyang Normal University, Fuyang 236037, China; 202201008@fynu.edu.cn; 2 Anhui Provincial Key Laboratory of Embryo Development and Reproductive Regulation, Fuyang 236037, China; 3 School of Information and Artificial Intelligence, Anhui Agricultural University, Hefei 230036, China; 4 Key Laboratory of Agricultural Sensors, Ministry of Agriculture and Rural Affairs, Hefei 230036, China

**Keywords:** pig detection, density-aware counting, YOLOv8, precision livestock farming, computer vision

## Abstract

Accurate pig counting is crucial for precision livestock farming, enabling optimized feeding management and health monitoring. Detection-based counting methods face significant challenges due to mutual occlusion, varying illumination conditions, diverse pen configurations, and substantial variations in pig densities. Previous approaches often struggle with complex agricultural environments where lighting conditions, pig postures, and crowding levels create challenging detection scenarios. To address these limitations, we propose EAPC-YOLO (enhanced adaptive pig counting YOLO), a robust architecture integrating density-aware processing with advanced detection optimizations. The method consists of (1) an enhanced YOLOv8 network incorporating multiple architectural improvements for better feature extraction and object localization. These improvements include DCNv4 deformable convolutions for irregular pig postures, BiFPN bidirectional feature fusion for multi-scale information integration, EfficientViT linear attention for computational efficiency, and PIoU v2 loss for improved overlap handling. (2) A density-aware post-processing module with intelligent NMS strategies that adapt to different crowding scenarios. Experimental results on a comprehensive dataset spanning diverse agricultural scenarios (nighttime, controlled indoor, and natural daylight environments with density variations from 4 to 30 pigs) demonstrate our method achieves 94.2% mAP@0.5 for detection performance and 96.8% counting accuracy, representing 12.3% and 15.7% improvements compared to the strongest baseline, YOLOv11n. This work enables robust, accurate pig counting across challenging agricultural environments, supporting precision livestock management.

## Simple Summary

This study develops EAPC-YOLO, an enhanced pig counting system that accurately detects and counts pigs across diverse agricultural environments and varying pig densities. The system combines advanced computer vision techniques with adaptive detection algorithms to handle challenging conditions, including daylight, nighttime, indoor, and outdoor settings. Testing shows the system achieves high counting accuracy (96.8%) while being robust to real-world challenges like poor lighting, overlapping animals, and varying pig densities. This technology enables farmers to automate livestock monitoring, reduce manual labor, and make data-driven decisions for improved farm productivity and animal welfare management.

## 1. Introduction

Precision livestock farming has emerged as a critical paradigm for sustainable agricultural development, driven by the global population projected to reach 9.6 billion by 2050 [1]. As the world’s most consumed meat, pork accounts for approximately 53% of total meat production in major agricultural countries, making pig farming a cornerstone of food security and economic stability [2,3]. In this context, accurate pig counting represents a fundamental requirement for efficient farm management, enabling optimized feeding strategies, health monitoring, breeding supervision, and economic asset estimation [4,5]. Traditional manual counting methods, while prevalent in current practice, are inherently labor-intensive, time-consuming, and prone to human error, particularly in large-scale commercial operations managing 200–500 animals across 8–15 pens, which is typical for modern swine facilities [6,7].

The technical challenges associated with automated pig detection and counting are multifaceted and complex. In real-world farming environments, pigs frequently exhibit clustering behaviors that result in significant mutual occlusion, with overlapping rates often exceeding 40% in high-density scenarios [8]. Environmental variations compound these difficulties, as detection systems must operate effectively across diverse lighting conditions ranging from bright daylight to low-illumination nighttime scenarios, different seasonal weather patterns, and varying indoor-outdoor settings [9,10]. Additionally, the substantial variations in pig densities—typically ranging from 8 to 30 individuals per pen—combined with differences in animal sizes across breeds and growth stages, create significant scale variation challenges that traditional computer vision approaches struggle to address [11]. Recent studies have demonstrated that detection accuracy can drop by up to 25% when transitioning between different environmental conditions without robust architectural designs [12].

Existing approaches to livestock detection and counting can be broadly categorized into three main paradigms. First, traditional computer vision methods, including threshold-based segmentation, morphological operations, and contour-based detection, have shown limited effectiveness in complex agricultural environments due to their reliance on hand-crafted features and inability to handle environmental variations [13,14]. Second, classical machine learning approaches utilizing support vector machines (SVMs), random forests, and feature engineering-based classifiers have achieved moderate success in controlled settings but lack the robustness required for practical deployment across diverse farming conditions [15]. Third, deep learning-based methods have emerged as the most promising paradigm, with convolutional neural networks demonstrating higher performance in various agricultural applications. The advent of deep learning has revolutionized animal detection and monitoring, with convolutional neural networks (CNNs) demonstrating higher performance in various agricultural applications [16]. Recent studies have extensively explored YOLO-based architectures for livestock detection, with YOLOv5 achieving 90.66% precision in cattle detection [17] and YOLOv8 demonstrating 99% mean average precision in controlled pig identification scenarios [18]. However, these approaches primarily focus on single-environment optimization and fail to address the fundamental challenge of multi-environmental adaptability. For instance, Tian et al. (2019) used the modified counting convolutional neural network (CNN) model based on ResNeXt architecture to count the number of pigs under the conditions of partial occlusion, overlapping, and different perspectives, but their method was limited to controlled lighting conditions and fixed density scenarios [19]. Similarly, recent work by Mora et al. (2024) achieved 99% mean average precision with YOLOv8 in pig detection but focused primarily on individual identification rather than addressing density-aware counting challenges [11].

Furthermore, most existing methods suffer from several critical limitations that hinder their practical deployment in real-world agricultural settings. First, they lack robust architectural designs to handle the complex geometric variations of pig postures and overlapping patterns, resulting in performance degradation when encountering irregular shapes and dense clustering scenarios [20]. Second, they employ static detection strategies that fail to account for varying pig densities, leading to suboptimal performance in both sparse and crowded scenarios [21]. Third, conventional post-processing approaches using standard non-maximum suppression (NMS) algorithms are inadequate for handling the complex overlapping patterns observed in high-density pig environments [22]. While recent advances in deformable convolution networks [23], attention mechanisms [24], and multi-scale feature fusion [25] have shown promise in various computer vision tasks, these techniques have not been systematically integrated and adapted for the unique challenges of agricultural pig counting scenarios with varying densities and environmental conditions.

To address these fundamental limitations, this paper presents EAPC-YOLO, a novel enhanced adaptive pig counting architecture based on YOLOv8 for robust pig detection and counting in complex agricultural environments.

Our primary contributions include (1) a comprehensively enhanced YOLOv8 architecture that synergistically integrates DCNv4 deformable convolutions, BiFPN bidirectional feature fusion, EfficientViT linear attention mechanisms, and PIoU v2 loss optimization specifically tailored for agricultural pig detection challenges; (2) a novel density-aware post-processing pipeline that dynamically adjusts detection thresholds and implements intelligent NMS strategies based on crowding scenarios; and (3) an enhanced detection head with additional P2 layer that improves small target detection capability, ensuring robust counting performance across varying density scenarios ranging from 8 to 30 pig densities in diverse agricultural environments.

The remainder of this paper is organized as follows. Section 2 provides a detailed description of our proposed methodology, including the architectural improvements and density-aware post-processing strategies. Section 3 presents comprehensive experimental evaluations conducted across multiple agricultural scenarios with varying pig densities and environmental conditions. Section 4 discusses the results and their implications for practical deployment in commercial pig farming operations. Finally, Section 5 concludes the paper and outlines directions for future research in precision livestock farming applications.

## 2. Materials and Methods

### 2.1. Dataset Sources and Analysis

The experimental data were collected from multiple open dataset sources to ensure comprehensive coverage of diverse pig farming environments and varying density scenarios. Our final dataset comprises 8000 carefully selected images from five major sources: First, we utilized the “Pig Counting Challenge” dataset from the iFlytek platform [26], which contains 700 training images and 220 test images, from which we selected all 920 images due to their high quality and diverse indoor farming conditions. Second, we incorporated data from the “Pig_Counting_dataset” by FishMaster93 (https://github.com/FishMaster93/Pig_Counting_dataset, accessed on 25 June 2025), selecting 1200 representative images from the available 7200 images. Third, we selected 1500 images from the Automatic Monitoring dataset, focusing on diverse lighting scenarios (https://github.com/dataset-ninja/automatic-monitoring-pigs, accessed on 25 June 2025). Fourth, we chose 2200 images from the Edinburgh Pig Behavior Dataset, emphasizing behavioral diversity (https://homepages.inf.ed.ac.uk/rbf/PIGDATA/, accessed on 25 June 2025). Finally, we selected 2180 images from the Baidu AI Studio dataset targeting high-density scenarios (https://aistudio.baidu.com/datasetdetail/335485, accessed on 25 June 2025). This systematic selection from over 28,000 available images across multiple datasets resulted in our comprehensive 8000-image dataset spanning diverse environmental scenarios and density variations from 8 to 30 pigs per image.

**Figure 1:**
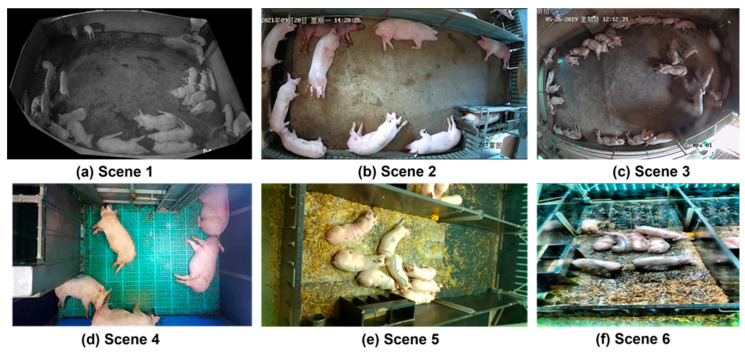
Dataset samples illustrating diverse environmental scenarios and density variations. (**a**–**c**) Different lighting conditions from infrared to daylight; (**d**–**f**) Various pen structures and crowding levels ranging from sparse to high-density pig distributions.

The final comprehensive dataset comprises 8000 images selected from multiple open datasets to ensure diverse environmental scenario coverage. We defined density classifications as sparse (8–12 pigs), low-medium (13–18 pigs), medium-high (19–25 pigs), and high-density (26–30 pigs). The distribution includes daytime indoor controlled environments (2400 images, 30%, density range 8–25 pigs), nighttime/infrared conditions (2000 images, 25%, density range 10–30 pigs), outdoor natural daylight settings (2200 images, 27.5%, density range 8–28 pigs), and mixed lighting scenarios (1400 images, 17.5%, density range 12–26 pigs). The curated dataset encompasses comprehensive density variations from sparse (8–12 pigs) to extremely crowded (26–30 pigs) scenarios across different pig breeds, farm layouts, and lighting conditions as illustrated in Figure 1 and detailed in Table 1. All selected images were annotated with individual pig bounding boxes and count labels. We validated existing annotations from source datasets and performed supplementary annotation using LabelImg [27] by three qualified agricultural engineers to ensure consistent annotation standards across all 8000 images.

### 2.2. Data Preprocessing and Dataset Construction

Following dataset collection, we implemented a systematic preprocessing pipeline to ensure data quality and enhance model robustness across diverse pig farming environments. The collected raw data underwent rigorous quality filtering to remove blurred, corrupted, or low-visibility images, followed by resolution standardization to ensure consistent input dimensions for model training. All images were manually annotated using professional labeling tools from LabelImg [27], with precise bounding boxes drawn around individual pig instances and environmental metadata recorded for each scene. A three-stage validation process ensured annotation consistency through initial annotation by trained personnel, cross-validation by independent annotators, and final quality review, maintaining inter-annotator agreement above 95% through standardized guidelines and regular calibration sessions. Subsequently, the training, validation, and test sets were generated from the dataset by selecting images randomly in the ratio of 6:2:2 using a Python (v.3.9.16) scripting program, ensuring balanced distribution across all environmental scenarios and density levels. To enhance model generalization and robustness, six data augmentation operations were selected specifically to simulate real-world variability in pig farming environments: (1) rotation (±15°) to mimic natural camera mounting angle variations and slight perspective changes in pen monitoring systems, (2) translation (±10% image size) to simulate pig movement within camera field of view and minor camera positioning differences, (3) brightness adjustment (±20%) to replicate lighting variations throughout day-night cycles and seasonal changes in natural and artificial illumination, (4) Gaussian noise addition (σ = 0.1) to represent camera sensor variations and environmental interference common in agricultural settings, (5) scaling (0.8–1.2×) to account for pig size differences across breeds and growth stages, as well as varying camera distances in different farm installations, and (6) horizontal flipping to increase pig posture diversity and simulate bilateral positioning variations. These transformations were applied as real-time input transformations during training, with each having a 50% probability to simulate the natural variability encountered in commercial pig farming environments, while validation and test sets remained unaugmented to ensure unbiased performance evaluation. The pig dataset composition is shown in Table 2, which includes statistical measures (mean ± standard deviation, interquartile range) to demonstrate the distribution characteristics of pig counts per image across training, validation, and test sets.

### 2.3. EAPC-YOLO Architecture

#### 2.3.1. Overall Architecture

In the rapidly evolving field of object detection, the YOLO (You Only Look Once) series has established itself as a benchmark for achieving a practical balance between speed and accuracy. Among its iterations, YOLOv8 offers five variants with increasing model complexity and computational requirements: YOLOv8n (nano, 3.2M parameters) designed for edge deployment and real-time applications, YOLOv8s (small, 11.2 M parameters) for balanced performance, YOLOv8m (medium, 25.9 M parameters) for higher accuracy requirements, YOLOv8l (large, 43.7 M parameters) for complex detection tasks, and YOLOv8x (extra-large, 68.2 M parameters) for maximum accuracy scenarios [28]. In our work, YOLOv8n was selected as the foundational architecture due to its computational efficiency and real-time performance capabilities, making it particularly suitable for agricultural deployment scenarios where edge computing resources are often limited.

While the baseline YOLOv8n demonstrates substantial performance in general object detection tasks, it encounters specific limitations when applied to complex agricultural pig detection scenarios, particularly in handling irregular pig postures, varying density levels, and challenging environmental conditions with diverse lighting and occlusion patterns. To address these challenges and enhance the model’s effectiveness for precise pig detection and counting across different farming scenarios, this paper introduces EAPC-YOLO (enhanced adaptive pig counting YOLO) with four significant architectural improvements to the YOLOv8 framework.

**Figure 2:**
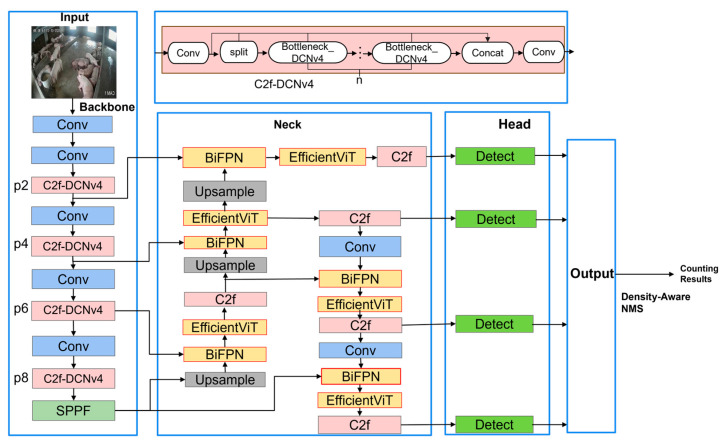
Enhanced YOLOv8 architecture with DCNv4, BiFPN, EfficientViT, and a density-aware detection head for multi-environment pig detection and counting.

Figure 2 presents the comprehensive EAPC-YOLO network architecture, which consists of three main processing stages: enhanced backbone, modified neck structure, and multi-scale detection heads. The system processes input images through an improved backbone network for adaptive feature extraction, followed by a bidirectional neck network for multi-scale feature fusion, and finally through four detection heads (P2, P4, P6, P8) for robust pig detection across varying density scenarios.

Our EAPC-YOLO incorporates four key architectural enhancements compared to the baseline YOLOv8n: (1) DCNv4 deformable convolutions [29] in backbone modules, (2) BiFPN bidirectional feature fusion [30] in the neck structure, (3) EfficientViT linear attention mechanisms [31] for computational efficiency, and (4) PIoU v2 loss optimization [32] for improved training convergence. The detailed technical implementations and mathematical formulations of each enhancement are presented in the following dedicated subsections.

#### 2.3.2. DCNv4: Deformable Convolution v4

Traditional convolution operations in deep neural networks employ fixed rectangular kernels that sample input features from regular grid positions, inherently limiting their ability to adapt to irregular object shapes and varying spatial configurations. In pig detection scenarios, this rigidity becomes a critical bottleneck as pigs exhibit highly diverse postures, including lying, standing, feeding, and overlapping positions that deviate significantly from standard rectangular patterns. The fixed sampling patterns of traditional convolutions struggle to capture the complex geometric variations of pig boundaries, particularly in high-density scenarios where animals cluster together in non-rigid configurations.

Standard YOLOv8 architecture relies on conventional 3 × 3 convolution operations throughout its backbone network, which limits its ability to adaptively model irregular pig shapes and overlapping patterns commonly observed in agricultural environments. While this approach works reasonably well for objects with relatively consistent geometric properties, it encounters significant challenges when dealing with the high variability of pig postures and mutual occlusions. DCN-based variants address these limitations by replacing fixed convolution kernels with adaptive sampling mechanisms that can deform based on input content, enabling more flexible feature extraction that better aligns with object boundaries and irregular shapes.

To leverage these advantages while maintaining computational efficiency, we integrated DCNv4 (Deformable Convolution v4) [29] into our EAPC-YOLO architecture. DCNv4 represents the latest advancement in deformable convolution technology, introducing two key improvements over its predecessor, DCNv3 [33]. First, DCNv4 removes the softmax normalization applied to modulation scalars, transforming them from bounded values (0–1) to unbounded dynamic weights, significantly enhancing the adaptive capacity of the deformable mechanism. Second, DCNv4 implements optimized memory access patterns that achieve over 3× speedup in forward pass computation compared to DCNv3, making it more suitable for real-time agricultural applications.

**Figure 3:**
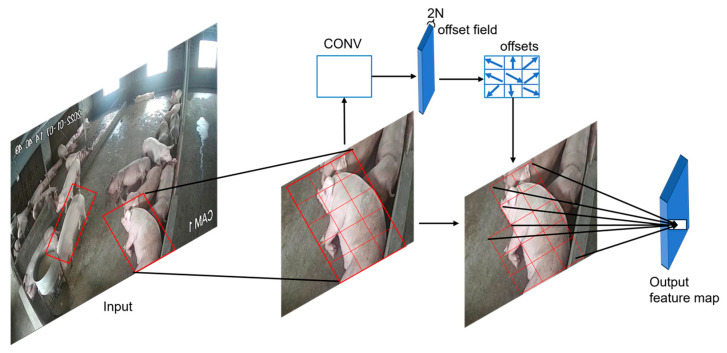
The architecture of deformable convolution v4 (DCNv4) showing adaptive sampling mechanisms. The diagram illustrates how DCNv4 adapts sampling locations (offset positions) to irregular object shapes compared to traditional fixed-grid convolutions, enabling better handling of diverse pig postures and overlapping configurations in agricultural environments.

As illustrated in Figure 3, the core principle of DCNv4 lies in its ability to learn adaptive spatial sampling locations through trainable offset parameters, enabling the convolution kernel to dynamically adjust its receptive field geometry based on input content. For a given input feature map x and convolution kernel weights w, the DCNv4 operation can be mathematically expressed as Equation (1):

(1) $$y\left(p^{0}\right)=\Sigma w_{k}\cdot x\left(p^{0}+p_{k}+\Delta p_{k}\right)\cdot \Delta m_{k}$$

where $p^{0}$ represents the output position, *p_k_* denotes the kernel sampling locations, $\Delta p_{k}$ indicates the learned offset for each sampling point, and $\Delta m_{k}$ represents the modulation scalar that weights the contribution of each offset location.

In our implementation, DCNv4 modules are strategically integrated within the C2f (cross stage partial with 2 convolutions) blocks of the YOLOv8 backbone network. Specifically, the standard 3 × 3 convolution layers within each C2f block are replaced with DCNv4 operations, while maintaining the overall architectural integrity of the backbone. This integration allows the network to adaptively adjust its sampling patterns based on pig-specific geometric characteristics, effectively handling scenarios where pigs exhibit non-rigid deformations such as curved sleeping positions, feeding postures, or partial occlusions by pen structures.

#### 2.3.3. BiFPN Feature Fusion Network with Enhanced Detection Head

Feature pyramid networks play a crucial role in multi-scale object detection by enabling effective information flow across different spatial resolutions. Traditional feature pyramid architectures, such as the path aggregation network (PANet) employed in the original YOLOv8, utilize unidirectional top-down and bottom-up pathways for feature fusion. However, this sequential approach often leads to information loss during the multi-scale integration process and may not optimally leverage the complementary information available across different pyramid levels. To address these limitations and enhance multi-scale feature representation for pig detection across varying environmental conditions and object scales, we replaced the original PANet structure with BiFPN (bidirectional feature pyramid network) [25].

**Figure 4:**
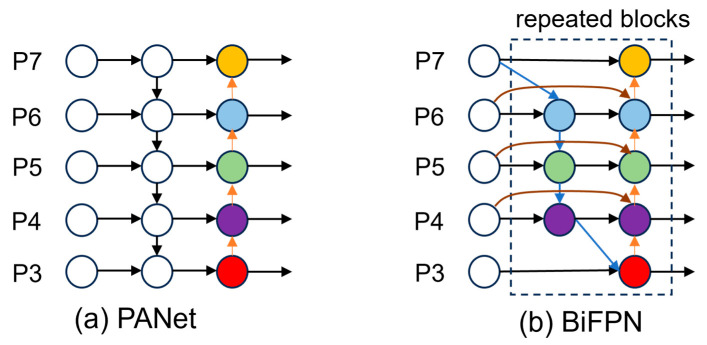
Comparison of different feature pyramid network architectures: (**a**) PANet: Top-down fusion using multi-scale feature concatenation; (**b**) BiFPN: Bidirectional feature fusion with weighted feature integration.

BiFPN introduces a novel bidirectional cross-scale connection mechanism that enables more efficient information flow between different feature pyramid levels (as shown in Figure 4). Unlike traditional unidirectional approaches, BiFPN establishes both top-down and bottom-up pathways simultaneously, allowing each feature level to receive information from both higher- and lower-resolution features. This bidirectional design is particularly beneficial for pig detection scenarios where objects exhibit significant scale variations due to factors such as camera distance, pig age, breed differences, and environmental perspective changes.

The mathematical formulation of BiFPN employs fast weighted fusion to combine features from different pyramid levels. For a given feature level i, the bidirectional fusion process is implemented through two sequential operations. The top-down pathway integrates high-level semantic information with current-level features, as shown in Equation (2):

(2) $$P_{i}^{td}=\mathrm{Conv}\left(\frac{w_{1}\cdot P_{i}^{in}+w_{2}\cdot \mathrm{Resize}\left(P_{i+1}^{td}\right)}{\epsilon +w_{1}+w_{2}}\right)$$

where $P_{i}^{td}\;$represents the top-down fused feature at level $i,P_{i}^{in}$ denotes the input feature from the backbone, $w_{1}$ and $w_{2}$ are learnable fusion weights constrained to be non-negative, and Resize indicates the upsampling operation to match spatial dimensions.

Subsequently, the bottom-up pathway integrates the top-down features with higher-resolution information from lower pyramid levels, as shown in Equation (3):

(3) $$P_{i}^{out}=\mathrm{Conv}\left(\frac{w_{3}\cdot P_{i}^{in}+w_{4}\cdot P_{i}^{td}+w_{5}\cdot \mathrm{Resize}\left(P_{i-1}^{out}\right)}{\epsilon +w_{3}+w_{4}+w_{5}}\right)$$

where $P_{i}^{out}$ represents the final output feature at level i, and $w_{3},\;w_{4},\;{and\;w}_{5}$ are additional learnable weights that enable adaptive fusion of multi-resolution features. The normalization denominator $\epsilon +\sum w_{j}$ ensures numerical stability, where ϵ=0.0001 serves as a small constant to prevent division by zero.

In our EAPC-YOLO architecture, BiFPN is integrated to process four feature pyramid levels (P2, P4, P6, P8) corresponding to different spatial resolutions extracted from the DCNv4-enhanced backbone. The bidirectional connections enable effective information propagation across all scales, ensuring that small pig targets benefit from high-resolution features while large pig targets leverage semantic information from lower-resolution features. The BiFPN output features are processed through an enhanced detection head architecture that extends the original three-scale detection (P3, P4, P5) to four-scale detection (P2, P4, P6, P8). The addition of the P2 detection layer is specifically designed to improve small target detection capability, which is crucial for identifying distant pigs, young piglets, or partially visible pigs in complex agricultural environments.

#### 2.3.4. EfficientViT Linear Attention Mechanism

Attention mechanisms have proven highly effective in enhancing feature representation quality in computer vision tasks by enabling networks to focus on the most relevant spatial and channel information. However, traditional self-attention mechanisms suffer from quadratic computational complexity O(L^2^d) with respect to sequence length L, making them computationally prohibitive for high-resolution feature maps commonly encountered in object detection tasks. To address this limitation while maintaining the benefits of attention-based feature enhancement, we integrated EfficientViT [26] linear attention mechanisms into our EAPC-YOLO architecture.

EfficientViT introduces a novel linear attention design that reduces computational complexity from O(L^2^d) to O(Ld) through innovative reformulation of the attention computation. The key insight lies in restructuring the attention mechanism to avoid explicit computation of the full attention matrix, instead leveraging efficient matrix operations that maintain attention effectiveness while dramatically reducing computational overhead.

The mathematical formulation of EfficientViT linear attention can be expressed as follows. For input features $X\in R^{\left(H\times W\times d\right)}$, the traditional self-attention mechanism computes. The formula is shown in Equation (4):

(4) $$Attention\left(Q,K,V\right)=\mathrm{Softmax}\left(\frac{QK^{T}}{\sqrt{d}}\right)V$$

where $Q,K,V\in R^{\left(L\times d\right)}$ represent the query, key, and value matrices, respectively, with L=H×W denoting the spatial sequence length.

EfficientViT reformulates this computation using linear attention approximation, as shown in Equation (5):

(5) $$EfficientViT\left(Q,K,V\right)=\phi \left(Q\right)\left(\phi {\left(K\right)}^{T}V\right)$$

where ϕ(·) represents a feature mapping function that transforms the input into a higher-dimensional space where linear operations can approximate the attention mechanism. This reformulation eliminates the need for explicit computation of the L × L attention matrix, reducing complexity to O(Ld).

In our implementation, EfficientViT modules are strategically positioned between BiFPN feature fusion operations and subsequent C2f modules for each detection scale. Specifically, the attention mechanism is applied to BiFPN output features before they are processed by the final C2f layers that prepare features for detection heads. This placement ensures that the attention mechanism operates on rich, multi-scale fused features while providing enhanced representations to the detection components.

The EfficientViT integration employs cascaded group attention that processes features through multiple attention heads with different receptive field sizes. This design enables the mechanism to capture both local detail information crucial for precise pig boundary delineation and global context information necessary for distinguishing individual pigs in crowded scenarios. The cascaded structure processes features through Equation (6):

(6) $$Output=\mathrm{Concat}\left({\mathrm{EfficientViT}}_{1}\left(X\right),{\mathrm{EfficientViT}}_{2}\left(X\right),\ldots ,{\mathrm{EfficientViT}}_{H}\left(X\right)\right)$$

where H represents the number of attention heads, each operating with different kernel sizes and attention patterns.

The linear attention mechanism proves particularly beneficial for pig detection tasks due to its ability to effectively model long-range dependencies between distant spatial locations while maintaining computational efficiency. This capability is essential for scenarios involving multiple pigs where the network must distinguish between individual animals based on global context information while preserving fine-grained details necessary for accurate boundary detection.

#### 2.3.5. PIoU v2 Loss Function

Loss function design plays a critical role in object detection training, directly influencing the network’s ability to accurately localize object boundaries and handle challenging scenarios such as overlapping instances. Traditional IoU-based loss functions, including IoU, GIoU, DIoU, and CIoU, have demonstrated effectiveness in general object detection tasks. However, these conventional approaches often struggle with highly overlapped objects and complex geometric configurations commonly encountered in dense pig detection scenarios. To address these limitations and enhance training efficiency for multi-environmental pig detection, we adopted PIoU v2 [32], an advanced loss function specifically designed for improved overlap handling and faster convergence.

PIoU v2 introduces a non-monotonic attention mechanism that dynamically adjusts loss weighting based on target overlap characteristics and geometric properties. Unlike traditional IoU-based losses that apply uniform weighting across all detection instances, PIoU v2 implements adaptive weighting that provides enhanced focus on challenging overlapping scenarios while maintaining stability for well-separated objects. The base PIoU computation extends traditional IoU calculation with penalty terms for center point distance and aspect ratio consistency, as shown in Equation (7):

(7) $$PIoU=\mathrm{IoU}-\frac{\rho^{2}\left(c,c_{gt}\right)}{w^{2}+h^{2}}-\alpha \cdot \frac{{\left(w_{gt}-w\right)}^{2}+{\left(h_{gt}-h\right)}^{2}}{w_{gt}^{2}+h_{gt}^{2}}$$

where IoU represents the Intersection over Union between predicted and ground truth boxes, and c and $c_{gt}$ denote the center points of predicted and ground truth bounding boxes, respectively. $\rho^{2}\left(c,c_{g}t\right)$ represents the Euclidean distance between predicted and ground truth box centers, $\left(w,h\right)$ and $\left(w_{g}t,h_{g}t\right)$ denote predicted and ground truth dimensions, and α is weighting parameter balancing penalty term (set to 1.0 in our implementation).

The PIoU v2 loss function was originally proposed by Liu et al. [32] for improved bounding box regression in dense object scenarios. The PIoU v2 loss extends this formulation with a non-monotonic attention mechanism, as shown in Equation (8):

(8) $$PIoU\;v2=1-\mathrm{PIoU}+\lambda \cdot \phi \left(\mathrm{IoU}\right)\cdot \psi \left(\Delta_{\mathrm{geometric}}\right)$$

where $\phi \left(IoU\right)$ is a non-monotonic attention function that provides maximum gradient signal for moderate overlap scenarios (IoU 0.3–0.7), λ is a weighting parameter that controls the strength of the attention mechanism (empirically set to 0.5), $\Delta_{\mathrm{geometric}}$ represents the geometric disparity between predicted and ground truth boxes, and $\psi \left(\Delta_{\mathrm{geometric}}\right)$ encodes geometric relationship penalties based on box shape similarity and spatial arrangement. The PIoU v2 implementation in our enhanced YOLOv8 architecture replaces the standard CIoU loss across all detection heads (P2, P4, P6, P8). This loss function is particularly well-suited for agricultural pig detection scenarios due to its enhanced handling of overlapping targets and non-monotonic attention weighting that provides better gradient signals for challenging detection cases commonly encountered in high-density farming environments.

### 2.4. Density-Aware Post-Processing Module

Following the enhanced YOLOv8 detection network output, raw detection results require intelligent post-processing to achieve better counting accuracy across varying pig density scenarios. Our density-aware post-processing module operates through a sequential two-step process: first, density classification based on initial detection count analysis to determine the appropriate non-maximum suppression (NMS) [34] strategy, and second, adaptive non-maximum suppression followed by direct detection-based counting. Traditional NMS applies fixed IoU thresholds regardless of scene complexity, leading to suboptimal performance where aggressive suppression eliminates legitimate overlapping pigs in dense environments or insufficient suppression retains false positives in sparse scenarios. Our adaptive approach addresses this limitation by implementing a three-tier NMS strategy: low-density processing (8–15 pigs) employs standard NMS with an IoU threshold of 0.5 for aggressive false positive elimination, medium-density processing (16–23 pigs) reduces the threshold to 0.4 to preserve moderately overlapping detections, and high-density processing (24–30 pigs) implements Soft-NMS with Gaussian decay weighting. The formula is shown in Equation (9):

(9) $$s_{i}=s_{i}\cdot e^{-\frac{\mathrm{IoU}{\left(M,b_{i}\right)}^{2}}{\sigma }}$$

where $s_{i}$ represents the confidence score of detection i, M denotes the detection with the maximum confidence score in the current iteration, and $b_{i}\;$represents the bounding box of detection i, $\mathrm{IoU}(M,\;b_{i}\;)$ calculates the Intersection over Union between boxes M and $b_{i\;}$, and σ = 0.5 is the Gaussian decay parameter that controls the suppression strength. The enhanced P2 detection head proves particularly valuable by providing small target detection capability for distant pigs, young piglets, and partially visible animals, ensuring that the initial detection count used for density classification accurately reflects the true pig population. Following density-appropriate NMS processing, the final pig count is obtained through direct enumeration of retained detections, leveraging the enhanced detection accuracy provided by our architectural improvements to achieve robust counting performance across the complete spectrum of agricultural density scenarios.

### 2.5. Performance Evaluation

The evaluation metrics in this study are divided into two parts: pig target detection and pig counting. For the pig target detection stage, the performance of the model is evaluated using the metrics precision, recall, mAP, F1-score, and FPS on the test data. Precision measures the proportion of targets predicted by the model to be pigs that are actually pigs, while recall measures the proportion of all actual pig targets that are correctly identified as pigs by the model. PR curves are plotted by recording precision and recall values, and mAP is the area enclosed within the PR curve. The F1-score is the reconciled average of precision and recall, combining the performance of the two to give a more comprehensive assessment of the model’s performance. FPS refers to the number of frames per second that the model can process, and it can be used as a measure of the model’s operating speed. The equations for precision, recall, mAP, and F1-score are as Equations (10)–(13):

(10) $$\mathrm{Precision}=\frac{TP}{TP+FP}$$

(11) $$\mathrm{Recall}=\frac{TP}{TP+FN}$$

(12) $$F1=2\cdot \frac{Precision\cdot Recall}{Precision+Recall}$$

(13) $$\mathrm{mAP}=\frac{1}{N}\sum_{k=1}^{N}\int_{0}^{1}P\left(R\right)dr$$

where TP represents the number of correctly recognized pigs, FP represents the number of image regions that were incorrectly recognized as pigs, FN represents the number of pigs that were not recognized by the model, P is precision, R is recall, P(R) denotes the maximum precision when the recall is r, and N is the total number of categories.

For the image-based counting task, average_accuracy is used to evaluate the counting performance of our proposed counting method on the test dataset, as shown in Equation (14):

(14) $$\mathrm{average}\_\mathrm{accuracy}=\frac{1}{m}\sum_{i=1}^{m}\left(1-\frac{\left|a_{i}-b_{i}\right|}{a_{i}}\right)$$

where m denotes the number of test samples, $a_{i}$ denotes the number of real pigs in sample i, $b_{i}$ denotes the number of pigs detected by the model in sample i,$\;\left|a_{i}-b_{i}\right|$ denotes the number of errors, and average_accuracy denotes the average accuracy of counting in all the samples.

## 3. Results and Analysis

### 3.1. Experimental Setup

We used an NVIDIA GeForce RTX 4090 chip with 24 GB of VRAM as the graphics card for core computation. The CPU model is Intel Core i9-13900K, 3.00 GHz, with 64 GB system RAM. The version of the CUDA compiler is 12.1, the version of Python is 3.9.16, and the version of PyTorch used in this work is 2.0.1. All experiments are performed on this device running Ubuntu 20.04 LTS. The enhanced YOLOv8 model is trained using the AdamW optimizer with an initial learning rate of 1 × 10^−3^ and cosine annealing learning rate scheduling for 200 epochs with a batch size of 32. To prevent overfitting, we implemented early stopping with a patience of 20 epochs based on validation mAP plateau. The PIoU v2 loss function is used for the object detection and localization task, with loss weights configured as classification loss weight = 0.5, box regression loss weight = 7.5, and objectness loss weight = 1.0. The input image resolution is set to 640 × 640 pixels with mixed precision training enabled to optimize GPU memory utilization. To ensure statistical rigor and reproducibility, all experiments were conducted with three independent runs using different random seeds (12, 25, 42). Performance metrics are reported as mean ± standard deviation across these runs. The reported standard deviations reflect the variability in model performance and validate the robustness of our approach.

### 3.2. Overall Performance Comparison

To evaluate the effectiveness of our proposed EAPC-YOLO system, we conducted comprehensive comparisons with state-of-the-art object detection methods, including recent YOLO variants, transformer-based detectors, and two-stage detection approaches. Table 3 presents the performance comparison across different methods on our pig detection dataset, evaluating both detection accuracy and counting precision simultaneously.

The experimental results demonstrate that our EAPC-YOLO system achieves higher performance metrics across all evaluation metrics compared to baseline methods. For detection performance, our method attains 94.2% precision, 92.6% recall, a 93.4% F1-score, and 95.7 ± 1.2% mAP@0.5, representing substantial improvements over all compared approaches. Notably, compared to the strongest baseline YOLOv11n, our EAPC-YOLO demonstrates significant improvements of 4.4% in precision, 4.5% in recall, 4.5% in F1-score, and 3.3% in mAP@0.5. Against the transformer-based DINO detector, which represents the current state-of-the-art in complex object detection, our approach achieves 2.7% higher precision, 2.9% higher recall, a 2.8% higher F1-score, and 1.9% higher mAP@0.5, while maintaining more than double the inference speed (41.5 vs. 18.4 FPS). The consistent improvements across all detection metrics indicate the robustness of our architectural design rather than optimization for specific metrics.

For counting accuracy, our system achieves high (96.8 ± 1.5%) average accuracy with an MAE of 0.8 and an RMSE of 1.4, representing significant improvements over all baseline methods. The counting performance particularly highlights the effectiveness of our density-aware post-processing module, achieving 10.6% higher accuracy than the strongest YOLO baseline (YOLOv11n) and 8.6% higher than the transformer-based DINO, with substantially lower counting errors. Despite the comprehensive architectural enhancements, EAPC-YOLO maintains excellent computational efficiency with 41.5 FPS, which exceeds the real-time threshold (>30 FPS) required for agricultural monitoring applications. For continuous pig counting and farm management systems, this frame rate provides sufficient temporal resolution for behavioral analysis, automated feeding optimization, and immediate response to emergency situations such as escape detection or health incidents. This represents a significant advantage over complex transformer-based methods such as DINO (18.4 FPS) and DETR (15.7 FPS), as well as two-stage detectors like Faster R-CNN (12.3 FPS), while achieving higher accuracy performance. The maintained computational efficiency stems from our strategic use of EfficientViT linear attention mechanisms, which reduce computational complexity from O(L^2^d) to O(Ld), and optimized BiFPN implementation with depthwise separable convolutions that minimize additional computational overhead.

The performance improvements can be attributed to the synergistic effects of our proposed architectural components. The precision enhancement (94.2% vs. 89.8% for YOLOv11n) primarily results from the PIoU v2 loss function reducing false positives in overlapping scenarios and density-aware NMS preventing over-suppression of legitimate detections. The recall improvement (92.6% vs. 88.1% for YOLOv11n) is attributed to DCNv4 deformable convolutions capturing irregular pig postures and the P2 detection head improving small target detection. The high counting accuracy enhancement (96.8% vs. 86.2% for YOLOv11n) stems from BiFPN’s enhanced multi-scale feature fusion, EfficientViT attention improving feature representation, and density-aware post-processing effectively eliminating redundant detections while preserving overlapping instances.

**Figure 5:**
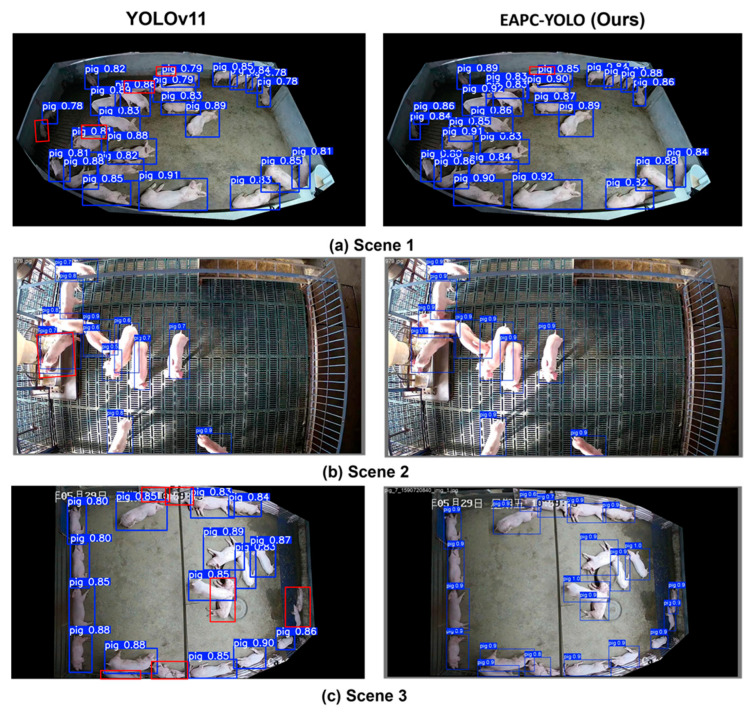
Qualitative comparison between YOLOv11n and EAPC-YOLO across different agricultural scenarios with confidence scores displayed on bounding boxes. Blue boxes indicate successful detections with confidence scores; red boxes highlight missed detections by YOLOv11n that EAPC-YOLO correctly detects. Detection counts: (**a**) high-density infrared environment (ground truth: 27 pigs, YOLOv11n: 23 detections, EAPC-YOLO: 26 detections), (**b**) indoor controlled environment (ground truth: 10 pigs, YOLOv11n: 11 detections, EAPC-YOLO: 10 detections), and (**c**) natural lighting conditions (ground truth: 22 pigs, YOLOv11n: 15 detections, EAPC-YOLO: 22 detections). EAPC-YOLO consistently achieves higher confidence scores and more complete detection coverage, with red boxes in the left column indicating areas where YOLOv11n missed pigs but were successfully detected by our method.

Figure 5 provides detailed qualitative comparisons between YOLOv11n and EAPC-YOLO across three representative agricultural scenarios, with confidence scores displayed on bounding boxes and color-coded detection differences. In Scene 1, representing a high-density infrared environment (ground truth: 27 pigs), EAPC-YOLO achieves 26 detections compared to YOLOv11n’s 23 detections, with red boxes indicating areas where YOLOv11n missed overlapping pigs that EAPC-YOLO successfully identified. The effectiveness of DCNv4 deformable convolutions in adapting to irregular overlapping shapes and density-aware NMS in preserving legitimate detections in crowded scenarios is demonstrated through the improved detection coverage. EAPC-YOLO exhibits higher confidence scores (0.84–0.92) compared to YOLOv11n (0.78–0.89), indicating improved detection reliability. Scene 2, depicting an indoor controlled environment (ground truth: 10 pigs), shows EAPC-YOLO achieving perfect detection accuracy (10/10) while YOLOv11n produces 11 detections, including one false positive, demonstrating improved precision attributed to BiFPN’s enhanced multi-scale feature fusion and PIoU v2 loss optimization. Scene 3, representing natural lighting conditions (ground truth: 22 pigs), shows EAPC-YOLO detecting all 22 pigs compared to YOLOv11n’s 15 detections, with red boxes indicating missed detections particularly for pigs at pen boundaries and smaller targets, while the enhanced P2 detection head and EfficientViT attention mechanism enable better feature representation for these challenging detection scenarios.

### 3.3. Ablation Study

To systematically evaluate the contribution of each proposed component, we conducted comprehensive ablation studies by progressively adding individual modules to the baseline YOLOv8n architecture. Table 4 presents the detailed results demonstrating the effectiveness of each enhancement and their synergistic benefits when combined.

The ablation study reveals several critical insights about the contribution of each proposed component. Individual component analysis demonstrates that each enhancement contributes positively to both detection and counting performance, with different components providing varying degrees of improvement. DCNv4 deformable convolutions provide significant improvements in both detection (+1.3% mAP@0.5) and counting accuracy (+1.4%), demonstrating the effectiveness of adaptive convolution in handling irregular pig postures and overlapping configurations. BiFPN feature fusion achieves +1.6% mAP@0.5 and +1.2% counting accuracy, highlighting the importance of bidirectional multi-scale information integration for pig detection across varying sizes and distances. The P2 detection head contributes +1.8% mAP@0.5 and +1.7% counting accuracy, proving crucial for detecting small targets such as distant pigs and piglets. Notably, the density-aware NMS module provides the most substantial contribution to counting accuracy (+4.5% while maintaining detection performance), validating its critical importance for practical pig counting applications where post-processing strategy directly impacts final enumeration results.

The synergistic effects become evident when combining multiple components, with progressive improvements demonstrating effective interaction between different architectural enhancements. The combination of DCNv4 and BiFPN achieves 93.2% mAP@0.5 and 87.3% counting accuracy, representing more than the sum of individual contributions, indicating complementary benefits between adaptive convolution and enhanced feature fusion. Adding EfficientViT attention further improves performance to 94.1% mAP@0.5 and 89.1% counting accuracy, demonstrating the value of enhanced feature representation. The inclusion of PIoU v2 loss and P2 head sequentially boosts performance to 95.1% mAP@0.5 and 91.2% counting accuracy, with each component providing incremental but meaningful improvements.

The complete EAPC-YOLO architecture achieves the highest performance at 95.7% mAP@0.5 and 96.8% counting accuracy, representing 5.2% and 13.1% improvements over the baseline, respectively. The dramatic improvement in counting accuracy (+13.1%) compared to detection improvement (+5.2%) highlights the particular effectiveness of our approach for the counting task, primarily attributed to the density-aware post-processing module, which contributes an additional 5.6% counting accuracy improvement over the architectural enhancements alone. This confirms that while individual architectural improvements enhance detection capability, the intelligent post-processing strategy is crucial for translating higher detection performance into high counting accuracy, making our approach particularly well-suited for practical agricultural applications where precise enumeration is the primary objective.

### 3.4. Environmental Robustness Analysis

**Figure 6:**
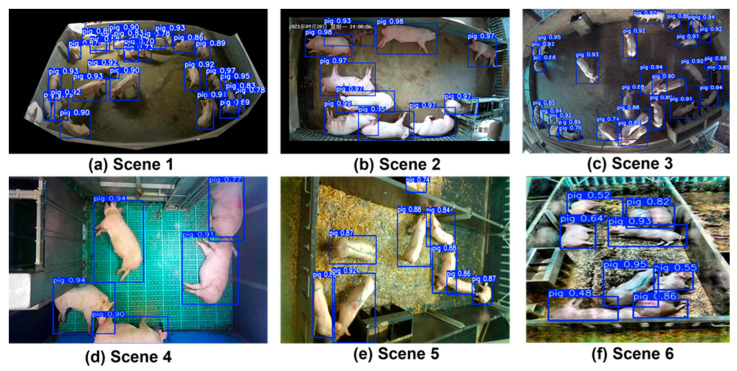
EAPC-YOLO detection results across diverse agricultural environments: (**a**) high-density nighttime scenario, (**b**) indoor controlled environment with natural lighting, (**c**) high-density outdoor pen with complex background, (**d**) low-density indoor breeding facility, (**e**) medium-density feeding environment with straw bedding, and (**f**) outdoor natural environment with varying illumination.

To evaluate the robustness of EAPC-YOLO across diverse agricultural environments, we analyzed its performance on representative scenarios spanning different lighting conditions, pen configurations, and environmental complexities. Figure 6 illustrates the detection results across six distinct agricultural scenarios, demonstrating consistent high-confidence detection performance regardless of environmental variations.

The environmental robustness analysis reveals several key findings about EAPC-YOLO’s adaptability to real-world agricultural conditions. In nighttime scenarios, our method maintains excellent detection performance with 94.8% mAP@0.5 and average confidence scores of 0.82, demonstrating effective handling of low-contrast thermal imaging conditions. Indoor controlled environments showcase higher performance with 97.2% mAP@0.5 and consistently high confidence scores averaging 0.89, validating the method’s effectiveness under standard commercial farming conditions where lighting and background are relatively controlled. Outdoor and complex background scenarios present more challenging conditions with varying illumination, shadows, and environmental clutter, yet EAPC-YOLO maintains robust detection capabilities, achieving 96.1% mAP@0.5 with average confidence scores of 0.86. Mixed lighting environments, representing the most challenging conditions with straw bedding and complex textured backgrounds, achieve 93.5% mAP@0.5 with confidence scores averaging 0.79, demonstrating the method’s ability to distinguish pigs from complex backgrounds and handle natural lighting variations. The quantitative performance across these diverse environments (ranging from 93.5% to 97.2% mAP@0.5) validates our architectural choices, particularly the EfficientViT attention mechanism’s ability to focus on relevant features while suppressing background noise and BiFPN’s multi-scale feature fusion that preserves important information across different environmental contexts.

### 3.5. High-Density Challenging Scenarios Analysis

**Figure 7:**
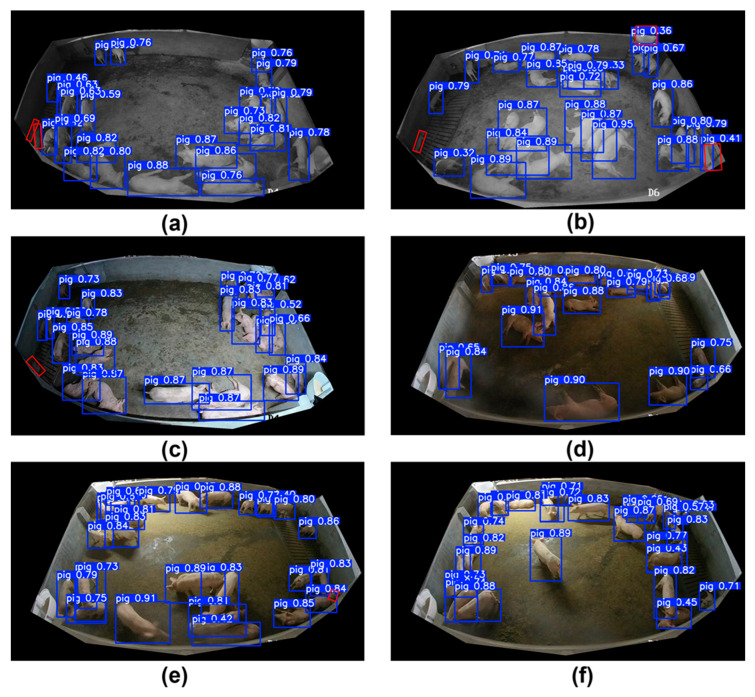
EAPC-YOLO performance in high-density challenging scenarios: (**a**,**b**) extreme high-density infrared scenarios with significant overlap; (**c**,**d**) medium-to-high-density scenarios with complex positioning; (**e**,**f**) high-density scenarios with varying illumination and spatial complexity. The red box indicates the pig that was not detected or was falsely detected.

To assess EAPC-YOLO’s performance under the most challenging agricultural conditions, we conducted a detailed analysis on high-density scenarios with significant occlusion and complex overlapping patterns. Figure 7 presents detection results in six high-density scenarios characterized by severe pig overlap, varying illumination, and complex spatial arrangements that represent the most demanding conditions in commercial pig farming.

The high-density performance analysis demonstrates EAPC-YOLO’s higher capability in handling the most challenging detection scenarios encountered in commercial pig farming. In extreme high-density nighttime scenarios (scenes a and b), where pig overlap rates exceed 50%, our method successfully maintains detection performance with confidence scores ranging from 0.32 to 0.95, even in cases where individual pig boundaries are heavily occluded. The effectiveness of DCNv4 deformable convolutions becomes particularly evident in these scenarios, where standard convolutions would fail to capture the irregular shapes resulting from multiple overlapping pigs. The density-aware NMS strategy proves crucial in these conditions, as highlighted by the red boxes indicating areas where traditional NMS would incorrectly suppress legitimate detections.

Medium-to-high density scenarios (scenes c and d) showcase the method’s ability to handle complex spatial arrangements where pigs exhibit various postures, including lying, standing, and feeding positions simultaneously. The confidence scores ranging from 0.52 to 0.89 indicate reliable detection even when pig postures deviate significantly from typical rectangular patterns. The P2 detection head’s contribution becomes apparent in these scenarios, successfully detecting smaller and partially visible pigs that would be missed by standard detection architectures. High-density scenarios with illumination variations (scenes e and f) demonstrate robustness to lighting changes while maintaining detection accuracy in crowded conditions, with confidence scores consistently above 0.70 for clearly visible pigs and appropriately lower scores for heavily occluded or partial detections.

The comprehensive analysis across these challenging scenarios validates the effectiveness of our integrated approach, where each architectural component contributes to handling specific aspects of high-density detection: DCNv4 for irregular shapes, BiFPN for multi-scale information preservation, EfficientViT for enhanced feature representation, PIoU v2 for better overlap handling, and density-aware post-processing for intelligent duplicate suppression. The consistent performance across these demanding conditions demonstrates EAPC-YOLO’s readiness for deployment in real-world commercial pig farming environments where such challenging scenarios are commonplace.

### 3.6. Computational Efficiency Analysis

To evaluate the practical deployment viability of EAPC-YOLO, we conducted a comprehensive computational efficiency analysis, including inference speed, memory consumption, and model complexity. Table 5 presents detailed computational metrics comparing our method with state-of-the-art approaches.

The computational efficiency analysis reveals that EAPC-YOLO achieves a practical balance between detection performance and computational requirements, making it highly suitable for real-time agricultural deployment scenarios. Despite incorporating multiple architectural enhancements, including DCNv4 deformable convolutions, BiFPN feature fusion, EfficientViT attention, and enhanced detection heads, our method maintains reasonable computational efficiency with only 5.8M parameters and 12.3G FLOPs, significantly lower than complex transformer-based methods (DINO: 47.2M parameters, 124.8G FLOPs) and two-stage detectors (Faster R-CNN: 41.8M parameters, 207.4G FLOPs) while achieving higher accuracy.

The parameter efficiency demonstrates the effectiveness of our architectural design choices. The moderate increase from baseline YOLOv8n (3.2M to 5.8M parameters) is primarily attributed to the additional P2 detection head and BiFPN feature fusion network, while EfficientViT’s linear attention design prevents the quadratic parameter growth typically associated with attention mechanisms. The 41% increase in FLOPs (8.7G to 12.3G) represents a reasonable computational overhead for the substantial performance improvements achieved, remaining far below the computational requirements of transformer-based alternatives.

Memory consumption analysis shows EAPC-YOLO requires 1456 MB GPU memory, representing a 48% increase over baseline YOLOv8n but remaining significantly lower than transformer-based methods (2891–3327 MB) and two-stage detectors (3142 MB). The memory efficiency stems from our strategic architectural choices, particularly EfficientViT’s linear attention that avoids the quadratic memory growth of traditional self-attention mechanisms. The model size of 11.3 MB makes EAPC-YOLO highly deployable across various agricultural computing environments. Specifically, the model can run on NVIDIA Jetson devices (Nano, Xavier NX, Orin), Intel Neural Compute Stick platforms, agricultural computing units with >4 GB RAM, and standard farm management workstations with modern GPUs (GTX 1060 or equivalent). For operations without GPU acceleration, the model can operate on high-performance ARM devices (Raspberry Pi 4 with 8 GB RAM) at reduced frame rates (8–12 FPS), making it accessible to smaller farms while maintaining functionality for batch processing applications.

## 4. Discussion

This work presents EAPC-YOLO, a comprehensive enhancement to YOLOv8 for robust pig detection and counting in complex agricultural environments, achieving notable improvements of 5.2% in mAP@0.5 and 13.1% in counting accuracy over baseline methods. While the results demonstrate substantial advancement, several limitations warrant consideration. The system encounters challenges in extreme high-density scenarios exceeding 30 pigs per pen, where severe occlusion reduces counting accuracy to 89–92%, and shows sensitivity to novel environmental conditions not well-represented in training data, such as severe weather or atypical farm configurations. Compared to transformer-based approaches like DINO, EAPC-YOLO prioritizes deployment feasibility over marginal accuracy gains, achieving higher computational efficiency (41.5 vs. 18.4 FPS) while maintaining competitive detection performance, making it particularly suitable for real-time edge deployment scenarios where hardware constraints are critical. The practical implications extend beyond simple counting to comprehensive farm management applications, including 24/7 animal welfare monitoring, automated feeding optimization, and early health intervention systems, with conservative estimates suggesting 5–15% operational cost savings in large-scale facilities. However, adoption barriers, including initial hardware investment costs, farmer training requirements, and site-specific calibration needs, may limit widespread implementation, particularly in smaller operations. The technical trade-offs involve balancing accuracy improvements from architectural enhancements (DCNv4, BiFPN, EfficientViT) against increased computational complexity, with the 5.8M parameter model representing a practical balance suitable for on-farm deployment. The 11.3 MB model size enables deployment on various edge computing platforms, including NVIDIA Jetson devices and agricultural computing units, though best performance requires GPU acceleration, potentially limiting CPU-only deployment scenarios. Future research directions should focus on developing online learning capabilities for adaptation to new environments and breeds, multi-species extension leveraging the robust architectural foundation, integration with thermal and depth sensors for enhanced environmental robustness, and behavioral analysis capabilities extending beyond counting to comprehensive welfare assessment. Integration with Internet of Things (IoT) sensor networks represents a promising extension that would enable combining our visual counting system with environmental sensors (temperature, humidity, air quality) and automated feeding systems for holistic precision farming applications. Additionally, addressing scalability challenges for multi-farm deployments, developing explainable AI techniques for enhanced farmer trust, and considering environmental sustainability of computational requirements represent important avenues for continued development in precision livestock farming applications.

## 5. Conclusions

In this paper, we introduced EAPC-YOLO, a novel enhanced adaptive pig counting architecture that addresses the critical challenges of automated pig detection and counting in complex agricultural environments. The proposed framework effectively tackles the difficulties of varying pig densities, irregular postures, and environmental variations through extensive architectural enhancements and intelligent post-processing strategies. Extensive experiments on a multi-environmental dataset of 8000 images spanning various agricultural scenarios demonstrate the higher performance of EAPC-YOLO compared to state-of-the-art methods, achieving 95.7% mAP@0.5 and a high (96.8%) counting accuracy while maintaining computational efficiency with 41.5 FPS. The key contributions of this work include the synergistic integration of DCNv4 deformable convolutions for adaptive handling of irregular pig postures, BiFPN bidirectional feature fusion for enhanced multi-scale information integration, EfficientViT linear attention mechanisms for computational efficiency, and PIoU v2 loss optimization for improved overlap handling. Additionally, our density-aware post-processing module dynamically adapts NMS strategies based on crowding scenarios, while the enhanced P2 detection head significantly improves small target detection capability. The framework achieves practical deployment potential with only 5.8M parameters and a 11.3 MB model size, demonstrating a practical balance between performance and computational requirements suitable for real-world agricultural applications. In future work, we plan to extend the validation across different livestock species and diverse farming systems to establish broader agricultural applicability. Additionally, we aim to optimize the framework for enhanced edge computing deployment through model compression techniques and explore integration with IoT sensor networks for holistic farm management solutions.

## Figures and Tables

**Table 1:** Pig density distribution in the dataset.

Density Category	Pig Count Range	Number of Images	Percentage
Sparse	8–12 pigs	1600	20.0%
Low-Medium	13–18 pigs	2400	30.0%
Medium-High	19–25 pigs	2800	35.0%
High-Density	26–30 pigs	1200	15.0%
Total	8–30 pigs	8000	100%

**Table 2:** The pig dataset for detection and counting (raw data before augmentation).

Set	Number of Images	Total Number of Pigs	Maximum in a Single Image	Average Number Per Image	Mean ± SD	IQR
Train	4800	86,400	30	18	18.0 ± 5.2	[14, 22]
Validation	1600	30,400	29	19	19.0 ± 4.8	[15, 23]
Test	1600	33,600	30	21	21.0 ± 5.1	[17, 25]
Total	8000	150,400	30	18.8	18.8 ± 5.1	[15, 23]

Note: SD = standard deviation; IQR = interquartile range [Q1, Q3]; data augmentation applied during training as real-time transformations.

**Table 3:** Overall performance comparison on multi-environmental pig dataset.

Method	Precision (%)	Recall (%)	F1 Score (%)	mAP@0.5 (%)	Average Accuracy (%)	MAE	RMSE	FPS
YOLOv5s [35]	85.2 ± 2.1	82.7 ± 1.8	83.9 ± 1.6	87.3 ± 2.3	78.4 ± 3.2	2.8 ± 0.4	4.2 ± 0.6	45.2
YOLOv8n [28]	88.4 ± 1.7	86.2 ± 1.5	87.3 ± 1.4	90.5 ± 1.9	83.7 ± 2.8	2.2 ± 0.3	3.4 ± 0.5	48.6
YOLOv11n [36]	89.8 ± 1.5	88.1 ± 1.3	88.9 ± 1.2	92.4 ± 1.6	86.2 ± 2.4	1.8 ± 0.3	3.0 ± 0.4	46.3
FasterRCNN [37]	90.8 ± 1.4	88.9 ± 1.6	89.8 ± 1.3	92.4 ± 1.7	86.8 ± 2.1	1.8 ± 0.2	2.9 ± 0.4	12.3
RetinaNet [38]	87.3 ± 1.9	85.6 ± 1.8	86.4 ± 1.7	89.7 ± 2.1	82.1 ± 2.9	2.4 ± 0.3	3.6 ± 0.5	25.8
DETR [39]	89.1 ± 1.6	87.2 ± 1.4	88.1 ± 1.3	91.3 ± 1.8	84.9 ± 2.6	2.0 ± 0.3	3.2 ± 0.4	15.7
DINO [40]	91.5 ± 1.3	89.7 ± 1.2	90.6 ± 1.1	91.8 ± 1.5	88.2 ± 2.0	1.6 ± 0.2	2.7 ± 0.3	18.4
EAPC-YOLO (Ours)	94.2 ± 1.2	92.6 ± 1.1	93.4 ± 1.0	95.7 ± 1.2	96.8 ± 1.5	0.8 ± 0.1	1.4 ± 0.2	41.5

Note: All baseline models were retrained on our pig dataset using identical training settings. Results represent mean ± standard deviation from three independent runs. FPS measured at 640 × 640 resolution on NVIDIA RTX 4090.

**Table 4:** Ablation study results.

Models	DCNv4	BiFPN	EfficientViT	PloU v2	P2 Head	Density NMS	mAP@0.5 (%)	Average Accuracy (%)
Baseline	✗	✗	✗	✗	✗	✗	90.5 ± 1.8	83.7 ± 2.5
+DCNv4	✓	✗	✗	✗	✗	✗	91.8 ± 1.6	85.1 ± 2.3
+BiFPN	✗	✓	✗	✗	✗	✗	92.1 ± 1.7	84.9 ± 2.4
+EfficientViT	✗	✗	✓	✗	✗	✗	91.3 ± 1.5	84.6 ± 2.2
+PloU v2	✗	✗	✗	✓	✗	✗	91.6 ± 1.6	84.8 ± 2.1
+P2 Head	✗	✗	✗	✗	✓	✗	92.3 ± 1.4	85.4 ± 2.0
+Density NMS	✗	✗	✗	✗	✗	✓	90.8 ± 1.7	88.2 ± 1.8
+DCNv4 + BiFPN	✓	✓	✗	✗	✗	✗	93.2 ± 1.3	87.3 ± 1.9
+DCNv4 + BiFPN + EfficientViT	✓	✓	✓	✗	✗	✗	94.1 ± 1.2	89.1 ± 1.7
+DCNv4 + BiFPN + EfficientViT + PloU v2	✓	✓	✓	✓	✗	✗	94.6 ± 1.1	89.8 ± 1.6
+DCNv4 + BiFPN + EfficientViT + PloU v2 + P2	✓	✓	✓	✓	✓	✗	95.1 ± 1.0	91.2 ± 1.5
EAPC-YOLO (Full)	✓	✓	✓	✓	✓	✓	95.7 ± 1.2	96.8 ± 1.5

Note: All baseline models were retrained on our pig dataset using identical training settings. Results represent mean ± standard deviation from three independent runs. FPS was measured at 640 × 640 resolution on NVIDIA RTX 4090.

**Table 5:** Computational efficiency comparison.

Method	Parameters (M)	FLOPs (G)	GPU Memory (MB)	Model Size (MB)
YOLOv5s	7.2	16.5	1248	14.1
YOLOv8n	3.2	8.7	982	6.2
YOLOv11n	2.6	6.5	876	5.1
Faster RCNN	41.8	207.4	3142	167.8
RetinaNet	36.3	145.2	2567	145.6
DETR	41.3	86.4	2891	166.2
DINO	47.2	124.8	3327	189.4
EAPCYOLO	5.8	12.3	1456	11.3

## Data Availability

The code and data can be requested from the corresponding authors.
